# Effect of supplementing chromium histidinate and picolinate complexes along with biotin on insulin sensitivity and related metabolic indices in rats fed a high‐fat diet

**DOI:** 10.1002/fsn3.851

**Published:** 2018-12-01

**Authors:** Cemal Orhan, Osman Kucuk, Mehmet Tuzcu, Nurhan Sahin, James R. Komorowski, Kazim Sahin

**Affiliations:** ^1^ Department of Animal Nutrition Faculty of Veterinary Science Firat University Elazig Turkey; ^2^ Department of Animal Nutrition Faculty of Veterinary Science Erciyes University Kayseri Turkey; ^3^ Division of Biology Faculty of Science Firat University Elazig Turkey; ^4^ Scientific and Regulatory Affairs Nutrition 21 Inc. Purchase NY USA

**Keywords:** biotin, chromium histidinate, chromium picolinate, GLUTs, high‐fat diet

## Abstract

**Scope:**

To investigate the effects of chromium histidinate (CrHis) and chromium picolinate (CrPic) complex along with biotin to a high‐fat diet (HFD) fed to rats on the insulin sensitivity and the anti‐obesity properties.

**Methods:**

Forty‐two Sprague–Dawley male rats were divided into six groups. The rats were fed either (a): a standard diet (Control) or (b): a HFD or (c): a HFD with biotin (HFD+B) or (d): a combination of HFD and biotin along with CrPic (HFD + B + CrPic) or (e): a combination of HFD and biotin along with CrHis (HFD + B + CrHis) or (f): a combination of HFD and biotin along with CrHis and CrPic (HFD + B + CrHis + CrPic).

**Results:**

Adding biotin with chromium to HFD improved the glucose, insulin, HOMA‐IR, leptin, lipid profile, with HFD+B+CrHis treatment being the most effective (*p* = 0.0001). Serum, liver, and brain tissue Cr concentrations increased upon Cr supplementations (*p *= 0.0001). Supplementing CrHis along with biotin to a HFD (HFD + B + CrHis) provided the greatest levels of GLUT‐1, GLUT‐3, PPAR‐γ, and IRS‐1, but the lowest level of NF‐κB in the brain and liver tissues.

**Conclusion:**

Biotin supplementation with chromium complexes, CrHis in particular, to a HFD pose to be a potential therapeutic feature for the treatment of insulin resistance.

## 1 INTRODUCTION

Nutrition plays a crucial role in the development of cancer, cardiovascular diseases, and diabetes (Rabhi, Hannou, Froguel, & Annicotte, 2017). Feeding a high‐fat diet (HFD) to experimental animals exerts a number of adverse metabolic alterations including hypertriglyceridemia, hyperinsulinemia, and glucose intolerance (Buchanan, Youn, Campese, & Sipos, 1992; Nascimento et al., 2013). A link between obesity, dyslipidemia, glucose intolerance, and hypertension has been proved, while insulin resistance and hyperinsulinemia have been implicated in the pathogenesis of multiple atherogenic risk factors (Slawson, Fitzgerald, & Morgan, 2013; Wang, Yuan, Duan, Li, & Hou, 2017). Hypertension and obesity as continuing challenges to public health efforts are major risk factors for cardiovascular morbidity and mortality. The brain is also often a target of diabetic complications such that prolonged hyperglycemic conditions cause a progressive impairment of neuronal function in the brain (Mooradian, 1997; Prasad, Sajja, Naik, & Cucullo, 2014).

The essentiality of chromium (Cr) has been questioned in the recent studies (Vincent, 2017). Although some studies suggested that chromium supplementation decreases insulin levels and improves glucose disposal rates in obese individuals (Cefalu et al., 1999; Talavera, Reza, & Cerda, 2004), in some other studies, Cr supplements to diabetic or healthy subjects did not clearly point out beneficial effects in glucose metabolism and diabetes (Bailey, 2014; Vincent, 2017). In contrast to the results from clinical works in humans, studies with rodent models supplemented with Cr have unambiguously indicated certain roles of Cr as a pharmacologically active element in glucose tolerance factor (Vincent, 2017). In this respect, supplementing chromium picolinate (CrPic) to the diet of obese rats has been shown to decrease plasma insulin, total cholesterol, and triacylglycerol concentrations as well as improved glucose disposal rates (Sahin et al., 2011; Wang, Zhang, Russell, Hulver, & Cefalu, 2006). The effects of supplementing different doses and the combination of chromium histidinate (CrHis) and CrPic along with biotin in rats fed HFD have not been reported. Therefore, the aim of this study were to investigate the effects of supplementing different complexes of CrHis and CrPic supplementation along with biotin on the insulin sensitivity and also to evaluate the anti‐obesity properties of these supplements through their action of mechanism by looking at the changes in biomarkers such as PPAR‐γ, IRS‐1, GLUTs, NF‐κB proteins, metabolic parameters, and tissue histopathological changes in rats fed HFD.

## 2 MATERIAL AND METHODS

### 2.1 Animals and diets

Sprague–Dawley male rats (*n* = 42, 8 weeks old) weighing 180–220 g were purchased from the Firat University Laboratory Animal Research Center (Elazig, Turkey). The animals were reared at the temperature of 22 ± 2°C, humidity of 55 ± 5%, and with a 12‐h light–12‐h dark cycle. All animal procedures were approved by the Animal Experimentation Ethics Committee of Firat University (Elazig, Turkey) (Bioethic Approval number 2014/17‐164). All procedures involving rats were conducted in strict compliance with the relevant laws, the Animal Welfare Act, Public Health Services Policy, and guidelines established by the Institutional Animal Care and Use Committee of the Institute.

### 2.2 Experimental design

After 1 week of adaptation period, the rats were divided according to BW, which was similar, into six equal groups containing seven rats each. The rats were fed either (a): a standard diet as control (Control) (12% of calories as fat) or (b): a HFD (42% of calories as fat) or (c): a HFD with biotin (300 μg/kg BW per d) (HFD + B) or (d): a combination of HFD and biotin along with CrPic (80 μg CrPic/kg BW per day) (HFD + B + CrPic) or (e): a combination of HFD and biotin along with CrHis (130 μg CrHis/kg BW per day) (HFD + B + CrHis) or (f): a combination of HFD and biotin along with CrHis (65 μg CrHis/kg BW per day) and CrPic (40 μg CrPic/kg BW per day) (HFD + B + CrHis + CrPic). Table 1 shows the composition of the control and the HFD fed to the rats. All chromium‐supplemented groups were provided approximately 10 μg/day elemental chromium. This amount was calculated based on 560 μg Cr that is needed for a 70‐kg adult human after adjusting doses based on metabolic body size (70^0.70 ^= 19.57 kg, needing 560 μg Cr; ~0.220^0.70 ^= 0.35 kg needing 10.02 μg Cr). The CrPic, CrHis, and biotin supplements were dissolved in drinking water and offered to rats via drinking water for 12 weeks. Cr‐chelates [Cr‐histidinate (CrHis) or Cr‐picolinate (CrPic)] and biotin were supplied by Nutrition 21, Inc. (Purchase, NY, USA).

**Table 1:** Ingredients of control and high‐fat diet (HFD) fed to rats

	Control diet^a^	High‐fat diet (HFD)^a^
Casein	200.0	200.0
Starch	579.5	150.0
Sucrose	50.0	149.5
Soybean oil	70.0	—
Beef tallow	—	400.0
Cellulose	50.0	50.0
Vitamin–mineral premix^b^	45.0	45.0
L‐cysteine	3.0	3.0
Choline bitartrate	2.5	2.5

a The control and HFD contained 0.786 ± 0.096 and 0.843 ± 0.055 mg Cr per kg diets.

b The vitamin–mineral premix provides the following (per kg): all‐*trans*‐retinyl acetate, 1.8 mg; cholecalciferol, 0.025 mg; all‐*rac*‐a‐tocopherol acetate, 12, 5 mg; menadione (menadione sodium bisulfate), 1.1 mg; riboflavin, 4.4 mg; thiamine (thiamine mononitrate), 1.1 mg; vitamin B + 6, 2.2 mg; niacin, 35 mg; Ca‐pantothenate, 10 mg; vitamin B + 12, 0.02 mg; folic acid, 0.55 mg; *d*‐biotin, 0.1 mg; manganese (from manganese oxide), 40 mg; iron (from iron sulfate), 12.5 mg; zinc (from zinc oxide), 25 mg; copper (from copper sulfate), 3.5 mg; iodine (from potassium iodide), 0.3 mg; selenium (from sodium selenite), 0.15 mg; choline chloride, 175 mg.

### 2.3 Laboratory analyses

At the end of the experiment, all rats were killed by cervical dislocation. Blood samples were taken from rats from the tail vein in the morning, after overnight fasting, and the tissues from the liver and brain were removed and processed for biochemical and Western blot examination. Fat was trimmed off from the slow‐twitch muscles (soleus and gastrocnemius deep portion). Visceral fat and liver weights were recorded. Initial body weight (BW), final BW, and feed intake were measured. Then, feed efficiency ratio (FER) was calculated as FER = [(total body weight gain × 100)/total feed intake].

Glucose, total cholesterol (TC), HDL cholesterol (HDL‐C), LDL cholesterol (LDL‐C), triglyceride (TG), free fatty acids (FFA), total protein (TP), total bilirubin (TBIL), blood urea nitrogen (BUN), and creatinine serum concentrations as well as alanine aminotransferase (ALT) and aspartate aminotransferase (AST) enzyme activities were measured by an automatic analyzer (Samsung LABGEO PT10; Samsung Electronics Co, Suwon, Korea). Repeatability and device/method precision of LABGEOPT10 was established according to the IVR‐PT06 Guideline. The concentration of serum leptin and insulin levels were measured with the rat leptin and insulin assay kit (Cayman Chemical Co., Ann Arbor, MI, USA) by ELISA (Elx‐800; Bio‐Tek Instruments Inc., Vermont, USA). The interassay and intra‐assay coefficients of variation were 4.6% and 6.3% and 3.8% and 5.5% for leptin and insulin, respectively.

Insulin resistance index was calculated by homeostasis model assessment of insulin resistance (HOMA‐IR) as (fasting glucose mmol/L) × (fasting insulin mU/L)/22.5. Because this calculation is human based, basal concentrations are not the same in the rodents and should be re‐estimated (strain differences) (van Dijk et al., 2013; Katz et al., 2000). Therefore, HOMA‐IR was calculated with a formula adapted to Matthews et al. (1985). For Sprague–Dawley male rats, reference values were calculated using average fasting glucose (5.1 mmol/L) and plasma insulin (43.9 mU/L) concentrations from all group (42 rats) at the beginning of the study (day 0). The HOMA‐IR score was calculated as the product of the fasting insulin level (mU/L) and the fasting glucose level (mmol/L), divided by 223.9 for rats. The cutoff value to define insulin resistance was HOMA‐IR ≥ 2.50. Rats presenting HOMA‐IR ≥ 2.50 were considered insulin‐resistant. Muscle malondialdehyde (MDA) concentrations were measured according to the previously described method (Akdemir et al., 2015) with a Shimadzu UV‐vis SPD‐10 AVP detector, a CTO‐10 AS VP column and 30 mM KH2PO4 and methanol (82.5: 17.5, v/v, pH 3.6) at a flow rate of 1.2 ml/min. Column waste was monitored at 250 nm.

Feed, serum, and tissue chromium concentrations were determined as described previously (Akdemir et al., 2015). For determination of Cr concentration, about 0.3 g feed, liver, and brain, as well as 0.5 ml serum samples were first digested with 5 ml concentrated nitric acid in a Microwave Digestion System (Berghof, Eningen, Germany) for 30 min. The specimens were subjected to graphite furnace atomic absorption spectrophotometer (AAS, Perkin‐Elmer, Analyst 800, Norwalk, CT, USA).

### 2.4 Western blot analyses

The protein levels of GLUT‐1, GLUT‐2, GLUT‐3, GLUT‐4, PPAR‐γ, IRS‐1, and NF‐κB in tissue were determined by Western blotting. To determine expressions of the brain and hepatic proteins in Western blot analysis, samples were homogenized in phosphate‐buffered saline (PBS) with protease inhibitor cocktail (Calbiochem, San Diego, CA, USA). The sample (20 μg of protein per lane) was mixed with sample buffer, boiled for 5 min, and separated by sodium dodecyl sulfate–polyacrylamide (12%) gel electrophoresis under denaturing conditions, and then electroblotted onto a nitrocellulose membrane (Schleicher and Schuell Inc., Keene, NH, USA). Nitrocellulose blots were washed in PBS and blocked with 1% bovine serum albumin in PBS for 1 h prior to application of the primary antibodies (GLUT‐1, GLUT‐2, GLUT‐3, GLUT‐4, PPAR‐γ, IRS‐1, and NF‐κB; Abcam, Cambridge, UK). Primary antibody was previously diluted (1:1000) in the same buffer containing 0.05% Tween‐20. The nitrocellulose membrane was incubated overnight at 4°C with protein antibody. The blots were washed and incubated with horseradish peroxidase‐conjugated goat antimouse IgG (Abcam). Specific binding was detected using diaminobenzidine and hydrogen peroxide as substrates. Protein load was controlled using a monoclonal mouse antibody against β‐actin antibody (Sigma, St. Louis, MO, USA). Protein levels were quantified densitometrically using an image analysis system (Image J; National Institute of Health, Bethesda, MD).

### 2.5 Statistical analysis

The alteration among groups was analyzed using one‐way analysis of variance (ANOVA) followed by the Tukey post hoc test (SAS Institute: SAS User's Guide: Statistics), and *p *<* *0.05 was considered statistically significant. Data were stated as a mean and standard error of the mean.

## 3 RESULTS

### 3.1 Body weight, visceral fat, and liver weights

Initial body weights, as planned, were similar among rat groups (*p *>* *0.05, Table 2). Feeding rats a HFD compared with control resulted in an increase in final BW, FER, and visceral fat as well as the liver weights but a decrease in feed intake (*p *=* *0.0001). However, supplementing biotin alone or combination of biotin with CrHis, CrPic, or CrHis + CrPic to HFD decreased the final BW, visceral fat, and the liver weights, with HFD + B + CrHis treatment having the lowest final BW (*p *=* *0.0001). However, adding any supplements to the HFD did not change feed intake or FER with the exception of biotin addition to HFD which decreased FER not as much as the other supplements (Table 2).

**Table 2:** Body weights and liver–visceral fat weight changes in rats fed a HFD supplemented with biotin and chromium complexes

Item	Treatments					
	Control	HFD	HFD + B	HFD + B + CrPic	HFD + B + CrHis	HFD + B + CrPic + CrHis
Initial BW, g	256.00 ± 5.03	256.29 ± 4.95	256.43 ± 7.56	256.86 + 6.45	256.29 ± 7.03	256.14 ± 8.09
Final BW, g	278.57 ± 1.65^d^	332.57 ± 2.35^a^	320.14 ± 1.40^b^	316.57 ± 1.19^bc^	310.29 ± 1.54^c^	314.57 ± 2.51^bc^
Feed intake, g/d	22.29 ± 0.59^a^	17.14 ± 0.59^b^	17.43 ± 0.75^b^	17.00 ± 0.62^b^	17.14 ± 0.70^b^	16.86 ± 0.51^b^
FER (g/100 g diet)	1.19 ± 0.16^c^	5.30 ± 0.06^a^	4.30 ± 0.28^ab^	4.14 ± 0.26^b^	3.99 ± 0.29^b^	3.76 ± 0.30^b^
Visceral fat, g	8.80 ± 0.18^e^	28.74 ± 0.38^a^	17.14 ± 0.38^b^	15.38 ± 0.23^dc^	15.16 ± 0.25^d^	16.59 ± 0.30^bc^
Liver weights, g	9.79 ± 0.29^c^	18.17 ± 0.28^a^	16.75 ± 0.44^ab^	16.31 ± 0.56^ab^	15.49 ± 0.62^b^	15.84 ± 0.66^b^

The data are presented as means and standard error. Means in the same line without a common superscript differ significantly (*p *<* *0.05).

B: biotin; CrPic: chromium picolinate; CrHis: chromium histidinate; FER: feed efficiency ratio = [(Total Body weight gain × 100)/total feed intake]; HFD: high‐fat diet.

### 3.2 Serum metabolites

**Figure 1:**
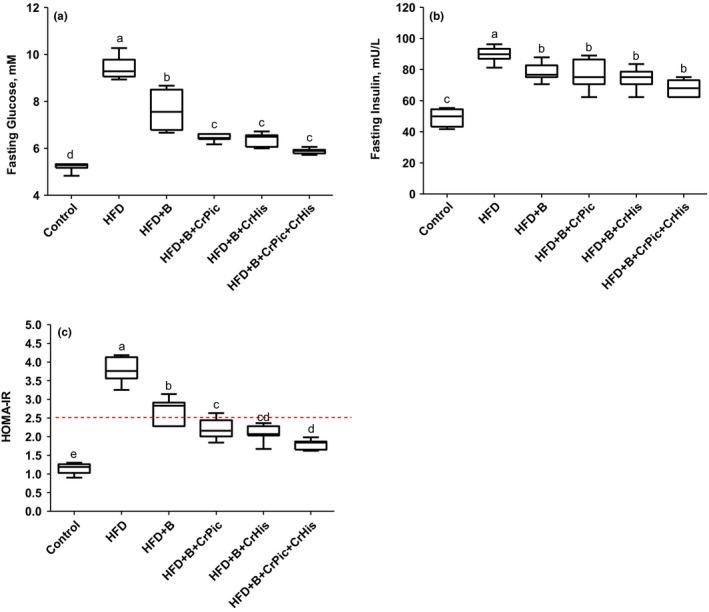
Effects of supplementing chromium histidinate (CrHis) and picolinate (CrPic) complexes along with biotin on plasma fasting glucose, insulin, and homeostatic model assessment insulin resistance (HOMA‐IR) in rats fed a high‐fat diet (HFD). Each plot represents the mean and standard error of the mean. Values within the plots with different superscripts are significantly different (Turkey's post hoc test, *p* < 0.05)

Feeding HFD to rats resulted in an increase in serum concentrations of glucose and insulin as well as HOMA‐IR index (*p *<* *0.05, Figure 1). Adding each of the supplements, but particularly Cr complexes, to a HFD decreased the serum concentrations of glucose, insulin, and HOMA‐IR index.

Changes in serum metabolites upon feeding HFD supplemented with biotin, CrHis, CrPic, and CrHis + CrPic are shown in Table 3. Feeding HFD to rats resulted in an increase in serum concentrations of FFA, leptin, TC, and TG as well as serum enzyme activities of AST and ALT (*p *=* *0.0001). Adding each of the supplements to a HFD decreased the serum concentrations and the enzyme activities. However, adding biotin and CrHis to HFD resulted in the lowest serum FFA, leptin, TC, and TG concentrations and serum AST enzyme activities (*p *=* *0.0001), bringing the concentration of TC and TG to base values similar to those of control. Feeding HFD alone or HFD with various supplements did not change the serum concentrations of HDL‐C (*p *=* *0.127; Table 3). However, feeding HFD to rats increased (*p *=* *0.0001) the serum LDL‐C concentrations which decreased with each of the supplement added to the HFD, being lowest with HFD + B + CrHis treatment (*p *=* *0.0001).

**Table 3:** Serum metabolite concentrations in rats fed a HFD supplemented with biotin and chromium complexes

Item	Treatments					
	Control	HFD	HFD + B	HFD + B + CrPic	HFD + B + CrHis	HFD + B + CrPic + CrHis
FFA, mM	2.62 ± 0.06^d^	4.22 ± 0.09^a^	3.49 ± 0.08^b^	3.37 ± 0.11^bc^	3.11 ± 0.05^c^	3.48 ± 0.06^b^
Leptin, ng/ml	73.86 ± 1.84^d^	220.41 ± 4.51^a^	188.57 ± 4.20^b^	186.71 ± 2.78^bc^	166.86 ± 9.02^c^	183.00 ± 3.34^bc^
TC, mg/ml	66.14 ± 6.98^c^	121.29 ± 6.78^a^	101.43 ± 4.57^ab^	93.71 ± 9.55^abc^	70.43 ± 6.12^c^	82.00 ± 3.68^bc^
HDL‐C, mg/dl	17.71 ± 0.99	14.00 ± 0.90	14.29 ± 1.48	14.71 ± 1.97	19.70 ± 2.57	15.84 ± 1.39
LDL‐C, mg/dl	24.86 ± 0.86^c^	60.71 ± 3.85^a^	48.43 ± 5.19^ab^	52.86 ± 7.64^ab^	41.14 ± 2.46^bc^	42.71 ± 2.70^abc^
TG, mg/dl	70.13 ± 4.61^d^	176.29 ± 12.09^a^	132.71 ± 8.37^b^	118.14 ± 10.69^bc^	72.57 ± 2.93^d^	93.29 ± 6.23^cd^
AST, U/L	120.57 ± 7.43^d^	301.00 ± 21.45^a^	255.43 ± 9.03^ab^	245.57 ± 13.98^ab^	184.57 ± 15.47^bc^	215.86 ± 31.89^b^
ALT, U/L	61.43 ± 2.86^b^	81.29 ± 4.26^a^	77.86 ± 3.23^a^	77.71 ± 7.40^a^	62.00 ± 6.86^b^	74.86 ± 3.97^ab^
TP, g/dl	6.38 ± 0.19	6.70 ± 0.35	6.76 ± 0.35	6.74 ± 0.28	6.51 ± 0.43	6.71 ± 0.26
TBIL, mg/dl	0.17 ± 0.01	0.22 ± 0.01	0.23 ± 0.01	0.21 ± 0.01	0.21 ± 0.02	0.23 ± 0.01
BUN, mg/dl	14.09 ± 1.17	14.79 ± 1.31	14.64 ± 1.12	14.04 ± 1.09	14.17 ± 1.10	14.57 ± 1.28
CRE, mg/dl	0.11 ± 0.01	0.13 ± 0.02	0.13 ± 0.02	0.11 ± 0.02	0.11 ± 0.02	0.13 ± 0.02

The data are presented as means and standard error. Means in the same line without a common superscript differ significantly (*p *<* *0.05).

AST: aspartate aminotransferase; ALT: alanine aminotransferase; B: biotin; BUN: blood urea nitrogen; CRE: creatinine; CrPic: chromium picolinate; CrHis: chromium histidinate; FFA: free fatty acid; HFD: high‐fat diet; HDL‐C: high‐density lipoprotein cholesterol; LDL‐C: low‐density lipoprotein cholesterol; TC: total cholesterol; TG: triglyceride; TP: total protein; TBIL: total bilirubin.

Adding biotin alone or biotin combination with chromium picolinate to HFD decreased the serum ALT activities in a similar pattern (77.86 and 77.71 U/L for HFD + B, HFD + B + CrPic, respectively). However, adding CrHis and a combination of CrHis and CrPic to HFD containing biotin further decreased the ALT enzyme activities, with HFD + B + CrHis treatment having the lowest values bringing the enzyme activities to base values similar to control. Serum TP, TBIL, BUN, and CRE concentrations remained similar among treatments (*p *≥* *0.06).

### 3.3 Oxidative stress indicator and Cr concentrations

As an indicator of oxidative stress, serum MDA concentrations increased with feeding HFD to rats (*p *=* *0.0001; Table 4). However, each treatment, namely, HFD + B, HFD + B + CrPic, HFD + B + CrHis, and HFD + B + CrHis + CrPic decreased the serum MDA concentrations with a similar magnitude. Although not significantly, feeding rats a HFD containing biotin and CrHis (HFD + B + CrHis) resulted in the lowest MDA concentrations.

**Table 4:** Serum MDA and tissue Cr concentrations in rats fed a HFD supplemented with biotin and chromium complexes

Item	Treatments					
	Control	HFD	HFD + B	HFD + B + CrPic	HFD + B + CrHis	HFD + B + CrPic + CrHis
Serum MDA, nmol/ml	1.011 ± 0.018^c^	2.201 ± 0.090^a^	1.573 ± 0.020^b^	1.526 ± 0.037^b^	1.396 ± 0.020^b^	1.484 ± 0.048^b^
Serum Cr, μg/ml	0.055 ± 0.002^b^	0.055 ± 0.003^b^	0.053 ± 0.005^b^	0.074 ± 0.001^a^	0.083 ± 0.001^a^	0.076 ± 0.003^a^
Liver Cr, μg/g wet tissue	0.641 ± 0.022^b^	0.638 ± 0.025^b^	0.676 ± 0.029^b^	0.948 ± 0.019^a^	1.058 ± 0.034^a^	0.967 ± 0.039^a^
Kidney Cr, μg/g wet tissue	0.619 ± 0.009^b^	0.620 ± 0.031^b^	0.671 ± 0.035^b^	0.906 ± 0.036^a^	1.059 ± 0.053^a^	0.944 ± 0.064^a^
Brain Cr, μg/g wet tissue	0.234 ± 0.009^c^	0.211 ± 0.013^c^	0.213 ± 0.012^c^	0.349 ± 0.013^b^	0.407 ± 0.012^a^	0.403 ± 0.008^a^

The data are presented as means and standard error. Means in the same line without a common superscript differ significantly (*p *<* *0.05).

B: biotin; CrPic: chromium picolinate; CrHis: chromium histidinate; HFD: high‐fat diet; MDA: malondialdehyde.

As expected, supplementing chromium complexes to the diet of rats, regardless of being fed with high fat or biotin, increased the serum, liver, and brain Cr concentrations (*p *=* *0.0001; Table 4). In this respect, supplementing all Cr sources responded to similar serum, liver, and kidney Cr concentrations. Although not significantly, serum and tissue Cr concentrations were greater with the treatment of HFD + B + CrHis.

Brain Cr concentration remained similar in rats fed either a control diet or HFD or HFD supplemented with biotin (*p *=* *0.0001). However, supplementing Cr complexes, as expected, increased the brain Cr concentration. Supplementing different Cr sources increased the brain Cr concentration in different magnitude, being more increases with the HFD + B + CrHis and HFD + B + CrPic‐CrHis treatments than that of HFD + B + CrPic.

### 3.4 Western blot analyses

**Figure 2:**
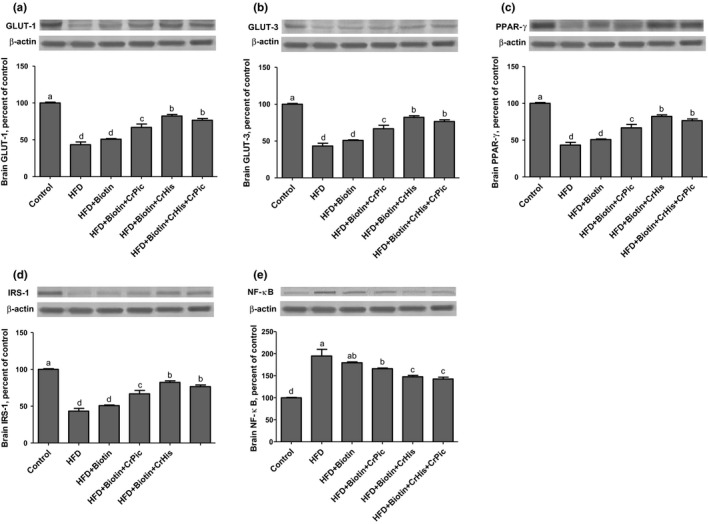
Effects of supplementing chromium histidinate (CrHis) and picolinate (CrPic) complexes along with biotin on (a) GLUT‐1, (b) GLUT‐3, (c) PPAR‐γ, (d) IRS‐1, and (e) NF‐κB protein levels in the brain of rats fed a high‐fat diet (HFD). Data are expressed as percent of the control value. Each bar represents the mean and standard error of the mean. Blots were repeated at least three times Western blot analysis was performed with actin included to ensure equal protein loading. The data are percentages of the control. Values within the bars with different superscripts are significantly different (Turkey's post hoc test, *p* < 0.05)

Brain GLUT‐1 and GLUT‐3 levels showed similar responses to the treatments (Figure 2 Panel a, b). The GLUT levels decreased in HFD‐fed rats compared to rats fed a control diet (100 vs. 40.99 and 30.37 for control, GLUT‐1, and GLUT‐3, respectively). Adding biotin and CrPic to HFD resulted in increases in the levels; however, adding CrHis and a combination of CrHis and CrPic further increased the levels with a similar magnitude (77.09 vs. 71.76 for GLUT‐1 and 73.94 vs. 68.04 for GLUT‐3, respectively).

Brain PPAR‐γ levels decreased in rats fed HFD diets compared to rats fed a control diet (100 vs. 43.38, Figure 2 Panel c). Brain PPAR‐γ levels increased upon feeding HFD supplemented with biotin and CrPic (76.62 vs. 60.50, respectively). Further increases in the expressions were observed with CrHis and a combination of CrHis and CrPic supplements. The levels were similar between HFD + B + CrHis and HFD + B + CrHis + CrPic treatments (89.97 vs. 86.96, respectively).

Feeding a HFD to rats resulted in a decrease in the brain IRS‐1 levels (Figure 2 Panel d). Although not significantly, adding biotin to the diet containing HFD increased the level (28.10 vs. 34.36). HFD + B + CrPic treatment resulted in increases in the levels (46.77); however, adding CrHis and a combination of CrHis and CrPic to HFD further increased the expressions in a similar magnitude (64.88 and 61.78 for HFD + B + CrHis and HFD + B + CrHis + CrPic, respectively).

Brain NF‐κB levels increased 243% in HFD‐fed rats compared with those of rats fed a control diet (Figure 2 Panel e). Adding biotin and CrPic to HFD resulted in decreases in the levels, but the levels were still greater than those of control (164.48 and 139.57 for HFD + B and HFD + B + CrPic, respectively). HFD + B and HFD + B + CrPic treatments influenced the levels in similarly. Adding CrHis and a combination of CrHis and CrPic to HFD further decreased the levels in a similar manner (87.75 vs. 102.85 for HFD + B + CrHis and HFD + B + CrHis + CrPic, respectively).

**Figure 3:**
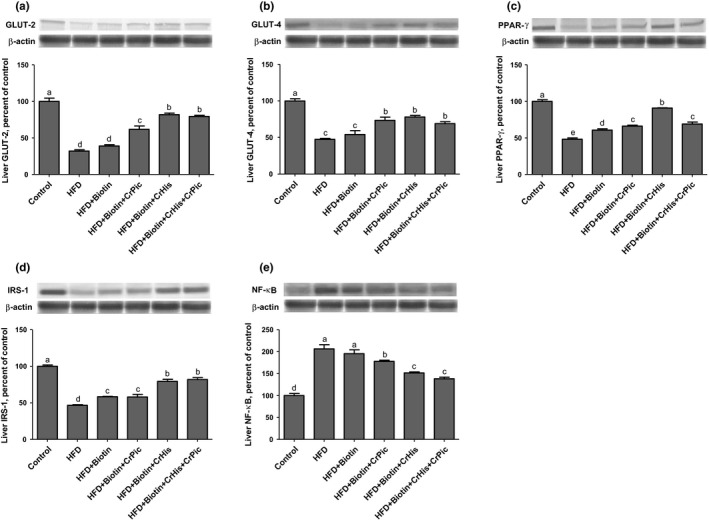
Effects of supplementing chromium histidinate (CrHis) and picolinate (CrPic) complexes along with biotin on (a) GLUT‐2, (b) GLUT‐4, (c) PPAR‐γ, (d) IRS‐1, and (e) NF‐κB protein levels in the liver of rats fed a high‐fat diet (HFD). Data are expressed as percent of the control value. Each bar represents the mean and standard error of the mean. Blots were repeated at least three times. Western blot analysis was performed with actin included to ensure equal protein loading. The data are percentages of the control. Values within the bars with different superscripts are significantly different (Turkey's post hoc test, *p* < 0.05)

The levels of GLUT‐2 and GLUT‐4 in the liver are shown in Figure 3 Panel a and b. The GLUT‐2 levels decreased upon feeding HFD diets in rats compared to a control diet‐fed rat (100 vs. 32.07). Adding biotin to a HFD increased the expression, but not significantly (39.02). Adding CrPic to HFD containing biotin resulted in significant increases in the levels (61.81). However, adding CrHis and a combination of CrHis and CrPic to HFD containing biotin further increased the levels in a similar magnitude (81.80 vs. 79.32 for HFD + B + CrHis and HFD + B + CrHis + CrPic, respectively).

The GLUT‐4 levels in the liver decreased in rats fed HFD compared to rats fed a control diet. Although not significantly, feeding a HFD supplemented with biotin increased the levels (47.41 vs. 54.04 for HFD and HFD + B, respectively). However, adding CrPic, CrHis and a combination of CrHis and CrPic to HFD containing biotin similarly increased the expressions, with being HFD + B + CrHis numerically greater (73.44, 78.04, and 68.91 for HFD + B + CrPic, HFD + B + CrHis, and HFD + B + CrHis + CrPic, respectively).

The rats fed HFD had a low expression of PPAR‐γ in the liver compared with rats fed a control diet (Figure 3 Panel c). Feeding HFD supplemented with biotin, CrPic, and CrHis resulted in significant increases in the levels (60.70, 66.11, and 90.93 for HFD + B, HFD + B + CrPic, and HFD + B + CrHis, respectively). However, supplementing a combination of CrHis and CrPic (HFD + B + CrHis + CrPic) resulted in a decrease in the levels (69.02) similar to the expression levels of HFD + B + CrPic.

Rats consuming HFD had lower liver IRS‐1 levels compared with the rats fed a control diet (Figure 3 Panel d). However, supplementing HFD with biotin and CrPic increased the levels (58.30 and 58.11 for HFD + B and HFD + B + CrPic, respectively), and further increases with similar magnitude were observed with CrHis and a combination of CrHis and CrPic supplementations (79.29 and 81.91 for HFD + B + CrHis and HFD + B + CrHis + CrPic, respectively).

The liver NF‐κB level decreased in rats fed HFD diets compared to rats fed a control diet (Figure 3 Panel e). Although not significantly, the level increased with the addition of biotin to HFD (28.67 vs. 36.02 for HFD and HFD + B, respectively). Adding CrPic to HFD resulted in significant increases in the levels (59.89). However, adding CrHis and a combination of CrHis and CrPic further increased the levels in a similar manner (80.89 vs. 78.28 for HFD + B + CrHis and HFD + B + CrHis + CrPic, respectively).

## 4 DISCUSSION

Feeding HFD to rats for 12 weeks increased the final BW, visceral fats, and liver weights 19.38%, 226.59%, and 85.60%, respectively. However, feed intake decreased with the same magnitude in all treatment groups. Decreased feed intake with feeding HFD could be due to an increase in serum leptin concentrations. In accord with the results of the present work, prolonged feeding with HFD has been shown to lead to obesity (Amin & Nagy, 2009; Buettner, Scholmerich, & Bollheimer, 2007; Tuzcu et al., 2011; Zhang, Lv, Li, Xu, & Chen, 2008). Obesity is a medical condition leading to serious health problems including heart disease, type 2 diabetes, certain types of cancer, osteoarthritis, and sleep disorders (Slawson et al., 2013). The treatment of obesity encloses the change of lifestyle including heart‐friendly eating and increased physical activity, and the use of authorities‐approved weight‐loss medicines and supplements. Biotin and chromium should be considered as supplements in this respect.

Biotin supplemented HFD fed to rats decreased final BW, visceral fats, and liver weights. The results from biotin supplementation were expected due to the fact that biotin functions as glucose and lipid homeostasis through regulating the expression of genes needed in the regulation of intermediary metabolism (Fernandez‐Mejia, 2005). Biotin has been found to stimulate the expression of insulin and pancreatic glucokinase, influencing insulin secretion (Romero‐Navarro et al., 1999), which were also the cases at the present work. Parallel to the results of the present work, biotin supplementation has been reported to ease the exacerbated hyperlipidemia (Báez‐Saldaña et al., 2004; Dukusova & Krivoruchenko, 1972). Marshall, Kliman, Washington, Mackin, & Weinland (1980) reported a negative correlation between biotin levels and total plasma lipids in healthy individuals. Zhang et al. (1996) also found that hyperglycemia is associated with biotin deficiencies in rats. In accord with the metabolic parameters measured at the present work, molecular parameters also supported the positive effect of biotin fed with HFD to rats for GLUT‐1, GLUT‐2, GLUT‐3, GLUT‐4, PPAR‐γ, IRS‐1, and NF‐κB levels in tissues.

The three different Cr sources, namely, CrPic, CrHis, and the combination of CrPic and CrHis, were supplemented at the present work. CrPic is known as the lipophilic or slow‐acting chromium complex, whereas CrHis is a hydrophilic or fast‐acting chromium complex absorbed more quickly than that of CrPic. The combination of CrPic and CrHis was a mix at the ratio of 1:1 for CrPic:CrHis. At the present work, supplementing CrHis along with biotin to HFD provided the best results yielding of the greatest levels of GLUT‐1, GLUT‐3, PPAR‐γ, and IRS‐1 but the lowest level of NF‐κB in the brain tissues, the greatest levels of GLUT‐1, GLUT‐3, and PPAR‐γ in the liver tissues, the lowest final body weights, visceral fat and liver weights, the lowest serum glucose, insulin, FFA, leptin, total cholesterol, LDL‐cholesterol, HDL‐cholesterol, and TG concentrations as well as lowest AST and ALT enzyme activities. Supplementing CrHis along with biotin to HFD also yielded second greatest levels of IRS‐1 and second lowest levels of NF‐κB in the liver tissues.

A wide range of metabolic dysfunctions characterized by insulin resistance, impaired glucose tolerance, obesity, intra‐abdominal adiposity, hypertension, dyslipidemia, increased inflammatory markers, and oxidative stress is called cardiometabolic syndrome. Such conditions as obesity, hyperlipidemia, and diabetes play a crucial role in the development of atherosclerotic cardiovascular diseases, potential causes of mortality and morbidity (Abdelaal, le Roux, & Docherty, 2017; Slawson et al., 2013). Chromium supplementation with CrPic, CrHis, or the combination (particularly CrHis alone) to HFD containing biotin resulted in an improvement in measured molecular parameters known to be characteristics to the conditions of cardiometabolic syndrome. Supplementing Cr with biotin to HFD also resulted in decreases in the final BW, visceral fats, and liver weights, promoting weight loss.

Rats fed HFD showed increased blood lipids and leptin concentrations which decreased by biotin and Cr supplementations, particularly CrHis. Circulating leptin concentrations increases in obesity. Therefore, the establishment of leptin resistance is considered as a major mechanism linking to the onset of obesity. Leptin is also crucial for the maintenance of glucose homeostasis and is considered as a potent insulin sensitizer (Yu, Park, Wang, Wang, & Unger, 2008). In accord with other parameters measured at the present work, supplementing biotin but particularly with CrHis improves insulin resistance as well as obesity.

Disorders in insulin signaling through disruption in lipid and glucose metabolism cause insulin resistance, an important characteristic feature of type 2 diabetes (Cordero‐Herrera, Martín, Bravo, Goya, & Ramos, 2013). Glucose is transported to the muscle cells via the insulin‐sensitive glucose transporter, GLUT4, and any disruption in the translocation of GLUT4 from an internal membrane pool to surface membranes may cause an impairment of insulin‐stimulated glucose uptake, eventually leading to insulin resistance (Huang & Czech, 2007). Chromium supplements attenuate insulin resistance by increasing the translocation of glucose transporters in tissues, accompanied by an increase in glucose uptake by the tissues. The effects of the Cr supplementations, CrHis in particular, in attenuating insulin resistance were supported at the present work by increased the brain GLUT1, GLUT3, the liver GLUT2, and GLUT4 levels.

Increased fasting glucose concentrations were parallel to the increases in the HOMA‐IR indexes in rats fed HFD. However, supplementations particularly Cr complexes decreased the index and the fasting glucose concentrations. These results once again highlight the importance of Cr in metabolic diseases.

The insulin receptor substrate‐1 (IRS‐1) is a key factor in insulin‐signaling pathways and thus in the development of type 2 diabetes. The IRS‐1 expression, previously decreased upon feeding HFD, increased with Cr complex supplementations, particularly CrHis, indicating the effects of Cr in improving insulin resistance by enhancing insulin signaling. Increased accumulation of fatty acids in tissues as seen in obesity leads serine/threonine phosphorylation of IRS proteins, resulting in a decreased signaling through reduced tyrosine phosphorylation and increased proteasomal degradation of IRS‐1 causing depression in the expression (Shulman, 2000; White, 2002). On the other hand, serine/threonine phosphorylation of IRS‐1 can also positively influence the insulin action via the residues which are specifically phosphorylated (Greene & Garofalo, 2002). A number of IRS1 kinases such as isoforms of PKC (protein kinase C), JNK (Jun N‐terminal kinase), IKK (IκB kinase), and p70S6K have been proposed to regulate the insulin action (Tanti et al., 2004).

In accord with the GLUT results, PPAR‐γ and IRS‐1 levels also responded to biotin and Cr supplementations in a similar way, improving insulin resistance. The peroxisome proliferator‐activated receptor (PPAR) family plays crucial roles in lipid metabolism, inflammation, glucose homeostasis, cell proliferation and differentiation, apoptosis, and aging (Bishop‐Bailey, 2000; Chinetti, Fruchart, & Staels, 2003; Howroyd, Swanson, Dunn, Cattley, & Corton, 2004). There are three isoforms of PPAR receptors that have specific, but also overlapping target genes, namely, PPAR‐α, PPAR‐γ, and PPAR‐β/δ. Activation of PPAR‐γ is known to lead insulin sensitization and to enhance glucose metabolism as well as to regulate adipocyte differentiation and fatty acid storage (Tyagi, Gupta, Saini, Kaushal, & Sharma, 2011). Decreased levels of PPAR‐γ in the brain and liver tissues of the rats fed HFD were soared to levels near that of control with supplementing Cr to HFD, indicating the effects of chromium in improving insulin resistance as well as obesity. PPAR‐γ activation is also known to inhibit the proliferation of malignant cells, including liposarcoma, breast adenocarcinoma, prostate carcinoma, colorectal carcinoma, non‐small‐cell lung carcinoma, pancreatic carcinoma, bladder cancer, gastric carcinoma, and glial tumors of the brain (Chattopadhyay et al., 2000; Rubin, Zhao, Kalus, & Simpson, 2000).

Nuclear factor‐κB (NF‐κB) is involved in cellular responses to stimuli such as stress, cytokines, free radicals, ultraviolet irradiation, and bacterial or viral antigens. Therefore, the role of NF‐kB in diseases such as inflammation, metabolic diseases including obesity, and cancer has been appreciated (Baker, Hayden, & Ghosh, 2011; Ben‐Neriah & Karin, 2011; Donath & Shoelson, 2011). A relationship between the activation of the transcription factor nuclear factor‐κB (NF‐κB) and fatty acid‐induced insulin resistance has been proposed (Kim et al., 2001). The expressions of NF‐κB at the present work increased in the brain and liver of rats fed a HFD. Supplementation of HFD with biotin and Cr reversed these responses (decreased). Qin et al. (2008) found that after a week of alcohol treatment in rats, IL‐10, an anti‐inflammatory cytokine produced by macrophages and lymphocytes that inhibit NF‐κB, increased in the liver but decreased in the brain.

Impairment of neuronal function as seen in stroke and cerebral ischemia are associated with diabetes along with prolonged hyperglycemia because of impaired cerebral vascular supply (Kissela et al., 2005; Li, Britton, Sima, & Dunbar, 2004; Mooradian, 1997; Saczynski et al., 2009). Chen et al. (2016) found that impairment of brain functions such as stroke in rats caused disturbance of tissue chromium homeostasis with a net loss through urinary excretion, and Cr supplementation restored tissue chromium levels attenuated post‐stroke brain infarction and hyperglycemia. In this respect, supplementation of Cr may have a potential effect on brain functions as also evidenced at the present work.

Accumulation of FFA and cholesterol may lead insulin resistance in the brain (Chaurasia & Summers, 2015; Chavez, Holland, Bar, Sandhoff, & Summers, 2005; De la Monte et al., 2010). Therefore, as evidenced in the present work as well, brain functions, high lipid profiles (obesity), and diabetes are all related to each other. The liver of rats fed a HFD showed an increased enzyme activity of ALT and AST as an indication of hepatic injury which was attenuated by biotin and Cr supplementation, particularly CrHis. Similar to the results of the present work, obese rats compared with normal rats (nonobese) were reported to have greater enzyme activities of ALT and AST (Kim, Ahn, Kim, & Jeong, 2016).

An increased level of serum MDA was observed in rats fed HFD, but biotin and chromium supplementations to the HFD decreased MDA levels. The results suggest that biotin and chromium have similar beneficial effects in attenuating oxidative stress caused by feeding a HFD. In accord with the results of the present work, it has been known that overproduction of reactive oxygen species (ROS) occurs in obesity caused by feeding a HFD (Furukawa et al., 2004; Zhang, Dong, Ren, Driscoll, & Culver, 2005), and the resulting oxidative damage leads to diabetes and related complications (West, 2000).

Improved parameters observed with Cr supplementation were due to the increased serum and tissue Cr concentrations, resulting in an improvement of insulin resistance as well as obesity. Elevated Cr concentrations of serum and the tissues in rats of the present work supplemented with Cr, CrHis in particular, were similar to those reported in different animal species with various doses (Anderson, Bryden, Polansky, & Richards, 1989; Wang & Xu, 2004; Wang et al., 2012; Zha, Wang, Xu, & Gu, 2007). Similar to the results from the present work, the apparent superiority of CrHis over CrPic was also supported by other studies in diabetic rats fed a HFD (Sahin et al., 2012; Tuzcu et al., 2011).

In conclusion, our results demonstrate that biotin supplementation alone or with chromium complexes, CrHis at particular, to a HFD pose to be a potential therapeutic feature for the treatment of insulin resistance and in the prevention of diabetes and its secondary complications.

## CONFLICT OF INTEREST

The authors declare that they do not have any conflict of interest. James R. Komorowski is employed by Nutrition 21, LLC., in Purchase, NY, USA.

## ETHICAL STATEMENT

All the animals received humane care according to the criteria outlined in the Guide for the Care and Use of Laboratory Animals prepared by the National Animal Research Committee and European Community (Directive 2010/63/EU). The study's protocols and procedures were ethically reviewed and approved by the Animal Experimentation Ethics Committee of University of Firat, Elazig, Turkey (animal ethics protocol no 2014/17‐164). Animal testing was necessary in this study.
